# Scaling properties of protein family phylogenies

**DOI:** 10.1186/1471-2148-11-155

**Published:** 2011-06-06

**Authors:** Alejandro Herrada, Víctor M Eguíluz, Emilio Hernández-García, Carlos M Duarte

**Affiliations:** 1 Instituto de Física Interdisciplinar y Sistemas Complejos, IFISC (CSIC-UIB), Campus Universitat de les Illes Balears, E-07122 Palma de Mallorca, Spain; 2 Instituto Mediterráneo de Estudios Avanzados, IMEDEA (CSIC-UIB), C/Miquel Marqués 21, E-07190 Esporles, Spain; 3 Oceans Institute, University of Western Australia, 35 Stirling Highway, Crawley 6009, Australia

## Abstract

**Background:**

One of the classical questions in evolutionary biology is how evolutionary processes are coupled at the gene and species level. With this motivation, we compare the topological properties (mainly the depth scaling, as a characterization of balance) of a large set of protein phylogenies with those of a set of species phylogenies.

**Results:**

The comparative analysis between protein and species phylogenies shows that both sets of phylogenies share a remarkably similar scaling behavior, suggesting the universality of branching rules and of the evolutionary processes that drive biological diversification from gene to species level. In order to explain such generality, we propose a simple model which allows us to estimate the proportion of evolvability/robustness needed to approximate the scaling behavior observed in the phylogenies, highlighting the relevance of the robustness of a biological system (species or protein) in the scaling properties of the phylogenetic trees.

**Conclusions:**

The invariance of the scaling properties at levels spanning from genes to species suggests that rules that govern the incapability of a biological system to diversify are equally relevant both at the gene and at the species level.

## Background

During the last century, an important effort has been devoted to the understanding of diversification patterns and processes in terms of branching evolutionary trees [1-7]. Tempo and mode of genetic change, and their connections with tempo and mode of speciation is an important issue in this context. In that sense, we address the question of whether similar forces act across the gene level and species-level evolution [8-10], through a comparative analysis of the topological behavior of protein and species phylogenies.

Previous analyses of the topological properties of phylogenies have revealed universal patterns of phylogenetic differentiation [3,6,7,11,12]. This means that the impact of evolutionary forces shaping the diversity of life on Earth on the shape of phylogenetic trees is, at least to the level of detail captured by the descriptors used, similar across a broad range of scales, from macro-evolution to speciation and population differentiation, and across diverse organisms such as eukaryotes, eubacteria, archaea or viruses, thereby. This together with the fact that evolutionary forces work at molecular level motivates the study of the topology of evolutionary relationships among molecular entities, looking for patterns of differentiation at such molecular level, thereby extending the examination of the universality of the scaling of branching laws in phylogenies all the way from molecular- to macro-evolution.

**Figure 1:**
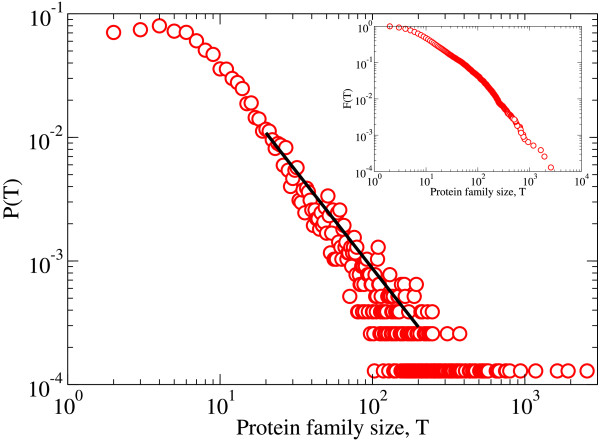
**Protein family size distribution**. Distribution of the size of the PANDIT protein families. Black line corresponds to a power law *P*(*T*) ~ *T*^-*γ*^, with a fitted exponent *γ *= 1.6 ± 0.1. The inset shows the complementary cumulative distribution *F *(*T*), that is, the probability of finding family sizes larger than *T*.

The term "protein family" was coined by Dayhoff in the 1960's to comprise similar proteins in structure and/or function, which are presumed to have evolved from a common ancestor protein [13]. Our analysis is based on a thorough data set of 7,738 protein families downloaded from the PANDIT database http://www.ebi.ac.uk/goldman-srv/pandit/[14] on May 27th 2008. It contains families with a broad range of sizes (see Figure 1). Taking into account that protein family diversification is driven by alternative evolutionary processes beyond speciation (orthology), such as gene duplication (paralogy), these data were used to test if the universal patterns found previously in species, subspecies, and higher taxonomic levels, also apply at the molecular evolutionary level. In particular we use tools derived from modern network theory [7,15-18] to examine the scaling of the branching in the protein family phylogenies.

A protein family phylogeny is represented as a tree, *i.e*., as an acyclic graph of nodes connected by branches (links), where each node represents a diversification event. For each node in a phylogeny, a subtree (or subfamily) *S *is made of the root at the selected node and all of its descendant nodes. The subtree size *A *is the total number of subfamily members that diversify from the root (including itself). The characterization of how protein diversity is arranged through the phylogenies can be achieved in a variety of ways [19-26]. We focus here on the *mean depth*, *d*, of the subtree *S *(see Methods) [6,27,28] defined as: , where, for a given node j, *d*_root,*j *_is its topological distance to the root of the subtree *S*, that is, the number of nodes one has to go through so as to go from that node to the root (including the root in the counting), and the sum is over all nodes in the subtree *S*. Note that we use here the mean depth over all subtree nodes and not just the leaves, which gives a different but related measure [4,29,30]. In the remainder, when no subindex is indicated, we understand that mean depth and other quantities refer to a whole tree or a subtree depending on the context.

How the shape of a phylogenetic tree, *i.e*., the distribution of protein diversification, changes with tree size, i.e., with the number of proteins it contains, can be analyzed by examining the dependence of the mean depth on subfamily size *d *= *d*(*A*). This gives information on the balance characteristics of the tree. To be clearer, in the additional file 1 we show the analysis of *A *and *d *for a fully balanced and a fully imbalanced 15-tip tree, as well as for a 15-tip subtree of a real phylogenetic tree. For a given tree size, the smallest value of the mean depth corresponds to the fully polytomic tree. The mean depth *d *as a function of tree size *A *is given in this case by(1)

For large sizes the leading contribution is *d*_min _~ 1. The largest mean depth value for a given size is given by the fully imbalanced, or asymmetric, binary tree with a mean depth given by(2)

which for large sizes *A *leads to the scaling behavior *d*_max _~ *A*. The fully balanced, or symmetric, binary tree is inside these extremes, with a mean depth given by(3)

The leading contribution at large sizes is logarithmic: d ~ ln *A*. This logarithmic scaling is not exclusive of fully balanced trees, it is also the behavior of the Equal-Rates Markov (ERM) model [28,31,32], the natural null model for stochastic tree construction, in which, at each time step, one of the existing leaves of the tree is chosen at random and bifurcated into two new leaves.

We report here the patterns of mean depth for protein families, and compare the branching patterns derived for protein families, from the PANDIT database with those of species phylogenies, reported previously from the TreeBASE database [7]. This comparison shows that branching patterns are mostly preserved across evolutionary scales spanning from genes to species.

## Results

### Protein phylogenies depth scaling

**Figure 2:**
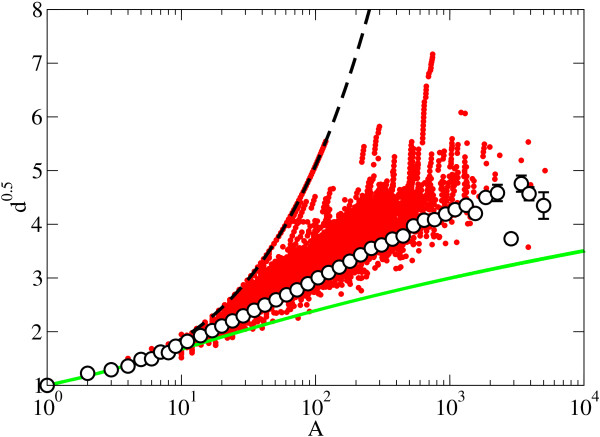
**Depth scaling of protein phylogenies**. Mean depth scaling for all protein families in the PANDIT database (solid circles, where each point represents a subtree) and the corresponding averaged binned depth (empty circles). Where the error bars are not visible we have that the standard error for the mean depth is smaller than the symbol size. The discontinuous and continuous lines correspond to the two extreme binary trees: fully imbalanced and fully balanced trees, respectively. The scales of the axes are chosen so that a behavior of the type d ~ (ln *A*)^2 ^appears as a straight line. Note that the values below the fully balanced tree scaling correspond to polytomic subtrees.

The analysis of the 7,738 protein phylogenies of PANDIT database shows (Figure 2) that the scaling of the mean depth with tree size lies between the two extreme topologies for binary trees (fully imbalanced and fully balanced trees), with the exception of a few polytomic subtrees, which display mean depth values lower that the one expected for the same size fully balanced binary tree. The data for independent protein trees are not scattered between the extreme cases but instead cluster in a space intermediate between these extremes depending on the size of the trees. Figure 2 displays depth, averaged within logarithmic bins of values of tree size *A*, as a function of *A*. The axes of this and other plots in the following have been chosen so that a depth behavior of the form *d *~ (ln *A*)^2 ^will appear as a straight line. This is the behavior suggested by the models in [33,34] and for organisms phylogenies in [6], which seems to correspond rather well to our data. The fully imbalanced tree shows a linear dependence *d *~ *A*, and the fully balanced tree shows a logarithmic dependence of the form *d *~ ln *A *(lines also shown in Figure 2).

**Figure 3:**
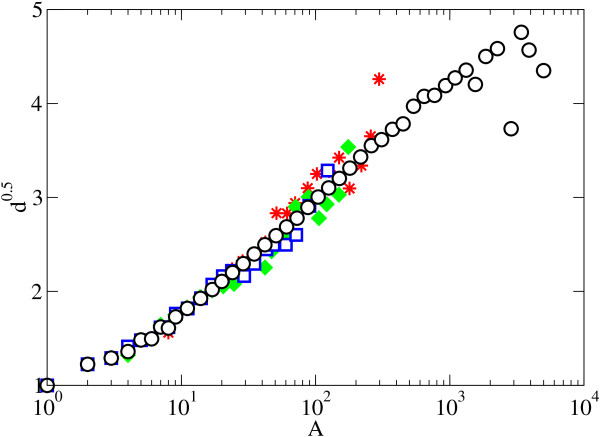
**Depth scaling of different protein functions**. Binned values of the mean depth for nuclear (empty squares), structural (solid diamonds) and metabolic (stars) protein families. The empty circles represent the averaged binned depth for the whole PANDIT database.

We analyzed the scaling of the mean depth as function of the tree size for different protein functions (e.g. nuclear, structural, metabolic) to assess whether different protein functions show scaling laws departing from the average mean depth scaling described for the whole PANDIT database. The results obtained show that the depth of different protein functions shows the same scaling with tree size as that described for the whole PANDIT dataset independently of function (Figure 3). This result supports the existence of universal scaling laws in the depth of protein phylogenies.

**Figure 4:**
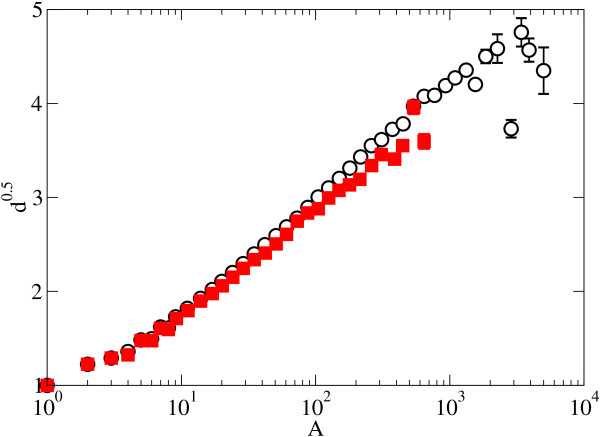
**Protein vs organism phylogenies**. Averaged and binned mean depth for organisms in TreeBASE (solid squares) and for protein phylogenies in PANDIT (empty circles). Where the error bars are not visible we have that the standard error for the mean depth is smaller than the symbol size.

The universality observed in the depth scaling of protein phylogenies is even more remarkable when protein phylogenies are compared with the species phylogenies [7] obtained from the TreeBASE database (Figure 4). The comparative analysis between PANDIT and TreeBASE shows a similar scaling of the mean depth with the tree size for both datasets. Although in a previous work with organism phylogenies the depth scaling was fitted to a power law [7], we find here that the squared logarithmic scaling d ~ (ln *A*)^2 ^of [6,33,34] provides also a reasonable fit for the protein families. Discriminating between these two scaling laws requires the comparison of larger trees, which are not available at the moment. Further discussion on this point is provided in the additional file 2. The important point, however, is that the analysis of protein phylogenies shows that the trees follow a scaling law as they speciate, which is universal across protein functions, and similar to that associated with the speciation at the species level.

**Figure 5:**
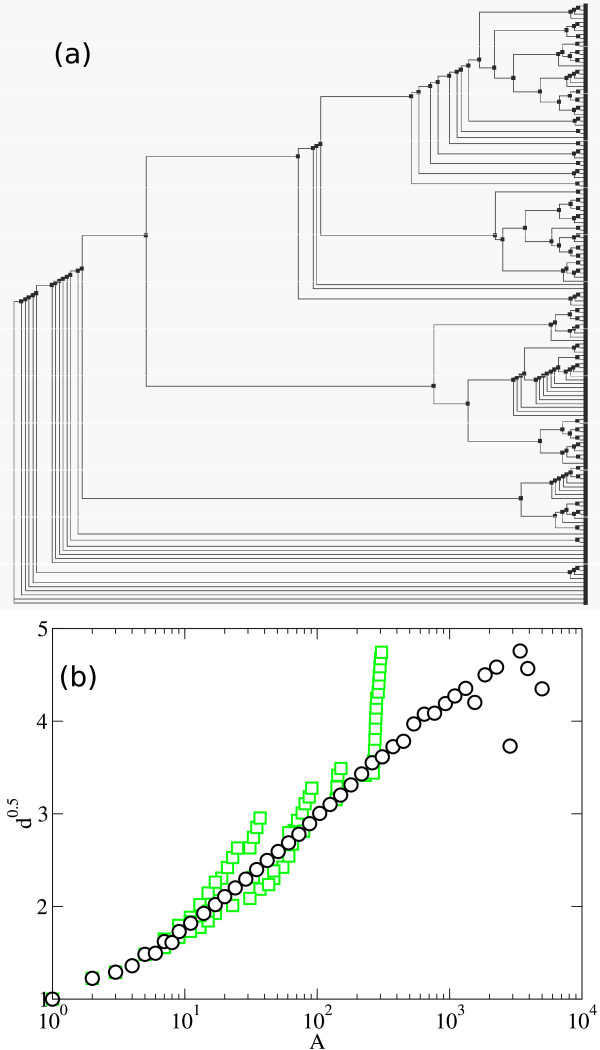
**Mean depth behavior for a specific phylogenetic tree**. (a) Phylogenetic tree corresponding to the Probable molybdopterin binding domain family (PF00994), with a high presence of imbalanced bifurcations close to the root. (b) Mean depth scaling of Probable molybdopterin binding domain family phylogenetic tree, where the empty squares correspond to the protein family. Solid circles represent the averaged and binned set for all the protein families of PANDIT.

There is some dispersion of the mean depth for the whole PANDIT dataset observed in Figure 2, which is attributable to imbalanced bifurcations in some specific trees. This increase in the presence of imbalanced bifurcations is reflected as a fast increase, characteristic of fully imbalanced trees. These regions with a high number of imbalanced bifurcations are most of the times close to the root, which can be related to a lack of resolution in the reconstruction process. In Figure 5 we show a detailed example of a phylogenetic tree with a region with a high presence of imbalance in the bifurcations close to the root, that leads to a dispersion from the mean depth scaling in the range A ∈ (2 × 10^2^, 3 × 10^2^), preserving the previously described universal mean depth scaling behavior in most of the size range, from 1 to 2 × 10^2^. The fact that the deviation from the mean is restricted only to certain regions of the phylogenetic trees, and that they do not affect significatively the average depth, thus preserving the global trend, supports the overall universality of the average depth scaling behavior found in the protein phylogenies from the PANDIT database.

### Evolvability model

The depth scaling behavior shared by protein and species phylogenies can be explained by different branching mechanisms. In this direction, during the last decade, several models have been published proposing different mechanisms to capture the topology of phylogenetic trees [6,27,28,33,35,36]. Most of the models proposed yield a logarithmic scaling of the mean depth, *i.e*., ERM-type for large sizes [31,32,37], which is not a good description of our data (see Figure 2 and additional file 2), at least at the tree sizes available; the AB model proposed in Ref. [33] is one of the few models that deviate from the ERM-like scaling leading to a squared logarithmic d ~ (ln *A*)^2 ^(see also [6]); models with power law scaling of the mean depth d ~ *A^η ^*have also been defined in terms of statistical rules assigning probabilities to different splittings or types of trees [33] or in terms of (simplified) evolutionary events (in the sense specified in Ref. [35]) occurring in time [27,28].

An alternative explanation of the scaling properties of the phylogenetic trees [36] suggests that the non-ERM behavior is a small-size transient behavior, which would cross-over to the ERM scaling d ~ ln *A *as larger tree sizes become available.

The process conducive to trees that deviate from ERM behavior is the presence of temporal correlations, which leads to asymptotic or just finite-size deviations with respect to the ERM behavior depending on whether these correlations are permanent or restricted to finite but large times. We, thus, explored the role of such correlations through a simple model based on the inheritability of the evolvability, *i.e*., the ability to evolve [38,39], as a biological characteristic which is itself inherited by sister species in speciation events. The process starts with the root, which we consider capable to speciate. At each time step, all present species capable to speciate branch simultaneously. Each branching event yields two new daughter species, for which we allow two possible outcomes:

• with probability *p*, the new species inherit the evolvability of the mother species, *i.e*., they have the same capacity as the mother species to speciate again;

• with probability 1- *p*, one of the daughter species is unable to speciate again, that is, only one of the two daughter species preserves the ability to evolve. Stemming from the definition of *robustness *as the property of a system to remain invariant in the presence of genetic or environmental perturbations [40], we consider a species' inability to speciate its robustness.

**Figure 6:**
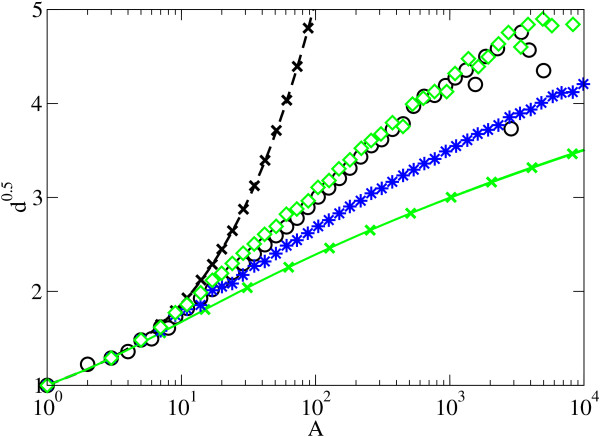
**Depth scaling of the evolvability model**. The mean depth scaling of the trees generated with the evolvability model for *p *= 1 and for *p *= 0 reproduces the mean depth scaling of the fully balanced (continuous line) and imbalanced binary trees (discontinuous line), respectively. The trees for *p *= 0.24 (empty diamonds) adjust the average behavior of protein (empty circles) very well. The stars correspond to trees for *p *= 0.5.

The first case gives rise to a symmetric speciation event, in which the two species emerging from the speciation event are similar, while the second one giving rise to asymmetries in the tree. If *p *= 1, we recover the completely balanced binary tree, while the topology obtained in the other extreme, *p *= 0, is the completely imbalanced binary tree (Figure 6). Thus the model combines symmetric with asymmetric branching introducing correlations (since one occurrence of the asymmetric event precludes further speciation on that branch), with the proportion determined by the parameter *p*.

The trees generated with this algorithm yield a scaling very close to those observed for phylogenetic trees in both PANDIT and TreeBASE for *p *= 0.24 (see Figure 6, and additional file 3). This result identifies the prevalence of imbalanced branching events (occurring with frequency 1- *p *= 0.76) relative to balanced ones (*p *= 0.24), which is consistent with earlier reports [5,6,33].

The correlations introduced by our model are not, however, permanent and ultimately a crossover to the random behavior appears for long sizes. To evaluate this, we calculated the analytical expression of the average depth, *d*. Taking into account that the expected number of o springs of a pair of sister nodes is 2*z *= 4*p *+ 2(1 - *p*) = 2(1 + *p*), starting with the root, the expected number of nodes after *n *branching events is(4)

where *z *= 1 + *p *is the expected o spring per sister node. The expected value of the cumulative branch size (see Methods) is given by(5)

At large *n*, the leading contributions are *A *~ *z^n ^*and *C *~ *nz^n ^*(we do not write explicitly prefactors which may depend on *z *but not on *n*). Taking into account Eq. (7) in Methods (i.e. *d *= (*C*/*A*) - 1) and inverting the relationship between *A *and *n *(*n *~ ln *A*), we obtain that for large sizes the leading order of the mean depth is *d *~ ln *A*, which indicates that what we observe in the simulations is a long transient behavior. This transient behavior leads to the fact that our model ts the proper behavior of the data at the sizes in the databases, but the asymptotic scaling at the larger sizes will finally be *d *~ ln *A*, as in the ERM.

## Discussion

The development of high-throughput "-omics" has provided the data required to address the traditional debate on how gene-level evolution shapes the species-level evolution [8-10]. This debate connects with that on the (dis)continuity between micro- and macro-evolution, and gradualism versus saltationism [41-43]. In the context of these debates, the universal scaling of phylogenetic trees at intra and inter-specific levels shown earlier [7] suggested the conservation of the evolutionary processes that drive biological diversification across the entire history of life. Here we extend this observation further to demonstrate that the universality of the scaling properties can also be extrapolated to the gene-level. The results presented here show that the branching and scaling patterns in protein families do not differ significantly from the patterns observed in species phylogenies, at least for the topological properties we have calculated. We do not observe any discrepancy between the shape of protein phylogenies and species phylogenies. Moreover, the results presented here shows no evidence for possible differences in phylogenetic trees among protein families with different biological functions, further providing evidence of universal, conserved evolutionary processes from genes to species.

In 2006, Cotton and Page published a comparative analysis between human gene phylogenies and species phylogenies [24]. They found quantitative differences between human paralogous gene and orthologous gene phylogenies. Their research focused on the comparison between (small) paralogous and orthologous gene families, while here we have analyzed complete protein families, which included both paralogous and orthologous protein members, focusing on the comparison between protein and species phylogenies. Our approach is based on a scaling analysis, examining how variables change with tree size, whereas the Cotton-Page's approach is based on a quantitative analysis of small sizes. This implies that despite their finding of quantitative differences between paralogous and orthologous gene phylogenies, we expect that both phylogenies would display scaling behavior similar to that we described here for complete protein phylogenies and organism phylogenies [7].

Different evolutionary models and mechanisms have been proposed to explain the branching patterns arising in evolution [6,27,28,33,36,37]. Here we have introduced a simple model accounting for differences in the degree of *evolvability*, which is emerging as a key trait constraint as important as robustness in evolution [44-47]. The model we proposed can be interpreted in the framework of the balance between evolvability as the potential of a biological system for future adaptive mutation and evolution [39], and robustness as the property of a system to produce relatively invariant output in the presence of a perturbation [40]. Indeed, the symmetric diversification event should correspond to the biological context in which the biological system is evolvable, while the asymmetric diversification process should correspond to a biological context where the new biological system, which has just appeared from the diversification process, is robust and unable of unlimited diversification.

The asymptotic behavior of our model at long tree sizes recovers the logarithmic behavior of the ERM scaling, so that, as in the models by [36], the non-ERM behavior occurs as a transient for the relatively small tree sizes present in the databases. Despite this, the local (i.e. present for finite sizes) imbalance in real trees can be interpreted in terms of the *evolvability *concept. The prevalence of the unbalanced branching found is consistent with previous works [6,33,48-51], and has been traditionally explained by the presence of variations in the speciation and/or extinction rates throughout the Tree of Life [4,5].

Different biological explanations for these variations in the speciation and/or extinction rates have been proposed, such as: refractory period [52], mass extinctions [53], specialization [4] or environment effects [54]. The consideration of an evolutionary scenario based on the evolvability/robustness interplay has led us to postulate the presence of asymmetric diversification events over the depth scaling during evolutionary processes giving rise to a new biological system which is unable to undergo a new diversification event. An incapability to diversify may occur at different levels of evolution, and can be found at the macroevolutionary level with taxa that require very long refractory periods or with random massive extinctions of taxa, as well as at the microevolutionary or gene level, where the elements unable to diversify are individuals from a population or genetic variants from a cell, embryo or individual.

## Conclusions

In summary, the finding of universal scaling properties at gene and species level, characterized by the similar scaling laws, strongly suggest the universality of branching rules, and hence of the evolutionary processes that drive biological diversification across the entire history of life, from genes to species. The topological characterization of phylogenetic trees has proven helpful to analyze the relevance of the robustness of a biological system (species or protein) in the scaling properties of the phylogenetic trees. Thus, the invariance of the scaling properties at levels spanning from genes to species suggests that the mechanisms leading to the incapability of a biological system to diversify for a very long period of time act at both the gene- and species-level.

## Methods

### 0.1 Protein phylogenies database

We analyzed the 7,738 protein families available in the PANDIT database (http://www.ebi.ac.uk/goldman-srv/pandit/ accession date May 27, 2008) [14]. PANDIT is based upon Pfam http://pfam.sanger.ac.uk/[55], and constitutes a large collection of protein family phylogenies from different signalling pathways, cellular organelles and biological functions, reconstructed with five different methods: NJ [56], BioNJ [57], Weighbor [58], FastME [59] and Phyml [60]. The size of each of the protein phylogenies, *T*, ranges from 2 to more than 2000 tips (i.e. proteins within families) and, in agreement with previous reports [20,61-64], shows a power law distribution *P*(*T*) ~*T*^-*γ *^(see Figure 1). Most of the bifurcations in these phylogenies are binary, with only 22% of polytomic bifurcations.

### 0.2 Mean depth

The definition of the mean depth *d *used here is directly related to the cumulative branch size [7,16-18,65] defined as . The sum runs over all nodes *j *in a tree and *A_j _*corresponds to the size of the subtree *S_j_*. The relationship between *C *and the mean depth can be obtained taking into account that the cumulative branch size can also be written as(6)

where *d*_root,*j *_is the distance of node *j *to the root. Thus, the mean depth of a tree is obtained as(7)

The depth of a tree can also be characterized by taking into account only the distance from the tips to the root. This is the case of the Sackin's index, *S*, which is defined as the sum of the depths of all the leaves of the tree [29]. Taking into account that a binary tree can be obtained as a growing tree adding at each time a speciation event we can calculate the change Δ*C *and Δ*S *at each speciation. If the distance of the node that speciates (leading to two new nodes) to the root is *d' *then(8)

while(9)

Accounting for the initial condition, that is, the root, with *C *= 1 and *S *= 0, yields *C *= 2*S *+ 1 for binary trees. Thus, at large sizes, both quantities, *C *and *S*, become proportional and scale in the same way with size.

## Authors' contributions

AH downloaded the protein phylogenies database, carried out the depth scaling analysis and designed the evolvability model. AH, VME and EHG designed the depth scaling analysis as well as provided the mathematical framework of the work. VME, EHG and CMD supervised the work. All authors participated in planning the work and writing the manuscript and read and approved the final manuscript.

## Supplementary Material

Additional file 1 **Branch size and mean depth examples**. The values of the branch size, *A *and of the mean depth, *d*, are shown (in brackets, as (*A*,*d*)) at each node of a fully balanced 15-tip phylogenetic tree (a), a fully imbalanced 15-tip phylogenetic tree (b), a 15-tip subtree of a real phylogenetic tree. Click here for file

Additional file 2 **Power-law *vs*. logarithmic scaling of the depth with tree size**. We compare the local exponents of the possible scaling laws of the depth with tree size for PANDIT. For sizes larger than 300 fluctuations make estimations unreliable. Filled squares: For the power-law scaling *d *~ *A^η ^*the local exponent at bin *i *is calculated as *η_i _*= Δ*_i _*ln *d*/Δ*_i _*ln *A*, where Δ*_i _*indicates the difference between two consecutive bins, for instance Δ*_i _*ln *d *= ln *d*(*i *+ 1) ln *d*(*i*). Empty diamonds: For the log scaling *d *~ (ln *A*)*^β ^*the local exponent at bin *i *is calculated as *β_i _*= Δ*_i _*ln *d*/Δ*_i _*ln ln *A*. Constant values of the local exponents, or values approaching a given value as sizes increase, indicate appropriateness of the corresponding scaling laws to describe the data. For the power-law scaling, the exponent is around *η *≃ 0.5 and slightly decays for larger trees. For the logarithmic scaling, the exponent approaches 2 as larger trees are considered, indicating *d *~ (ln *A*)^2^. The results indicate comparable quality of fit for both laws at the reliable range. Note that the simpler logarithmic law, *β *= 1, is not supported by the available data. Click here for file

Additional file 3 **Standard deviation of the evolvability model**. Values of the standard error (SE) of the results from simulations of the evolvability model with respect to the PANDIT dataset, for values of *p *between [0.21 - 0.27]. A value *p *= 0.24 minimizes the error. Click here for file
