# Supplementary material for: Scaling properties of protein family phylogenies
Source: BMC Evol Biol. 2011 Jun 6;11:155. doi: 10.1186/1471-2148-11-155 (PMC3277297; doi:10.1186/1471-2148-11-155)
Supplement: Additional file 1 — Branch size and mean depth examples. The values of the branch size, A and of the mean depth, d, are shown (in brackets, as (A,d)) at each node of a fully balanced 15-tip phylogenetic tree (a), a fully imbalanced 15-tip phylogenetic tree (b), a 15-tip subtree of a real phylogenetic tree. [file 1471-2148-11-155-S1.PDF]

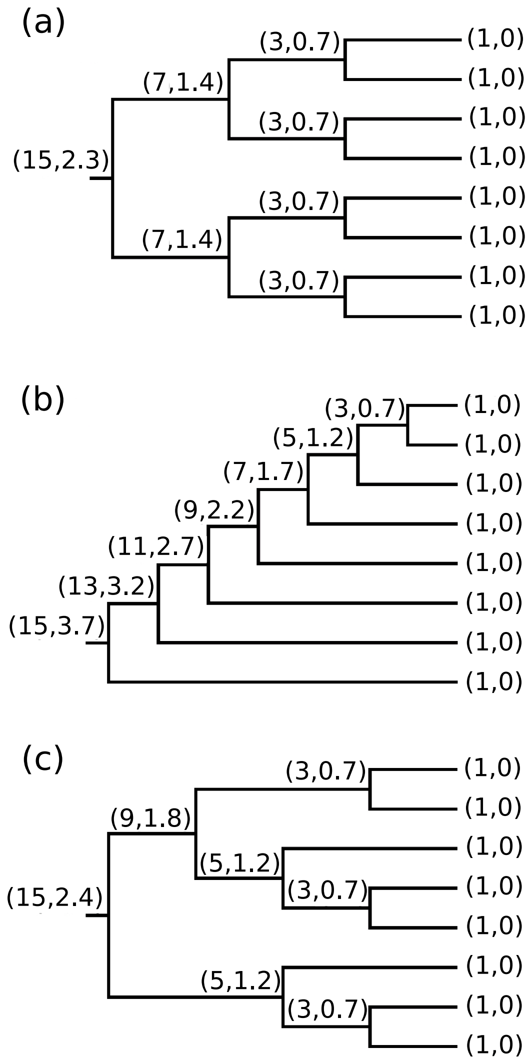

Additional file 1 — Branch size and mean depth examples.
The values of the branch size, A and of the mean depth, d, are shown (in brackets, as (A,d)) at each node of a fully balanced 15-tip phylogenetic tree (a), a fully imbalanced 15-tip phylogenetic tree (b), a 15-tip subtree of a real phylogenetic tree.
