# Supplementary material for: Scaling properties of protein family phylogenies
Source: BMC Evol Biol. 2011 Jun 6;11:155. doi: 10.1186/1471-2148-11-155 (PMC3277297; doi:10.1186/1471-2148-11-155)
Supplement: Additional file 2 — Power-law vs. logarithmic scaling of the depth with tree size. We compare the local exponents of the possible scaling laws of the depth with tree size for PANDIT. For sizes larger than 300 fluctuations make estimations unreliable. Filled squares: For the power-law scaling d ~ Aη the local exponent at bin i is calculated as ηi = Δi ln d/Δi ln A, where Δi indicates the difference between two consecutive bins, for instance Δi ln d = ln d(i + 1) ln d(i). Empty diamonds: For the log scaling d ~ (ln A)β the local exponent at bin i is calculated as βi = Δi ln d/Δi ln ln A. Constant values of the local exponents, or values approaching a given value as sizes increase, indicate appropriateness of the corresponding scaling laws to describe the data. For the power-law scaling, the exponent is around η ≃ 0.5 and slightly decays for larger trees. For the logarithmic scaling, the exponent approaches 2 as larger trees are considered, indicating d ~ (ln A)2. The results indicate comparable quality of fit for both laws at the reliable range. Note that the simpler logarithmic law, β = 1, is not supported by the available data. [file 1471-2148-11-155-S2.PDF]

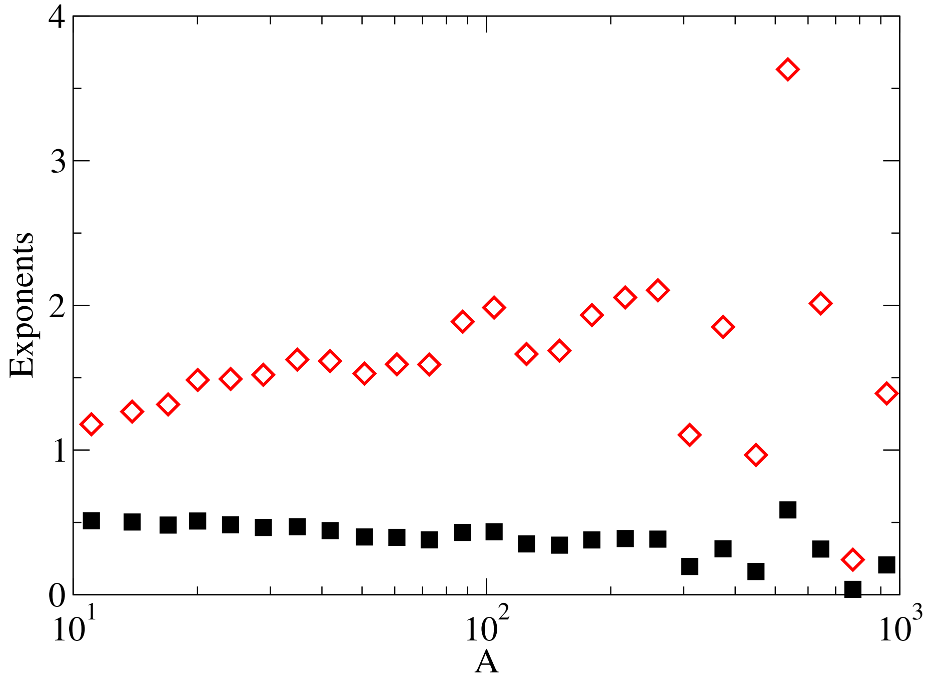

Additional file 2 — Power-law *vs.* **logarithmic scaling of the depth with tree size.**

We compare the local exponents of the possible scaling laws of the depth with tree size for PANDIT. For sizes larger than 300 fluctuations make estimations unreliable. Filled squares: For the power-law scaling $d \sim A^\eta$ the local exponent at bin i is calculated as $\eta_i = \Delta_i \ln d / \Delta_i \ln A$, where Δ_i indicates the difference between two consecutive bins, for instance $\Delta_i \ln d = \ln d(i+1) - \ln d(i)$. Empty diamonds: For the log scaling $d \sim (\ln A)^\beta$ the local exponent at bin i is calculated as $\beta_i = \Delta_i \ln d / \Delta_i \ln \ln A$. Constant values of the local exponents, or values approaching a given value as sizes increase, indicate appropriateness of the corresponding scaling laws to describe the data. For the power-law scaling, the exponent is around $\eta \simeq 0.5$ and slightly decays for larger trees. For the logarithmic scaling, the exponent approaches 2 as larger trees are considered, indicating $d \sim (\ln A)^2$. The results indicate comparable quality of fit for both laws at the reliable range. Note that the simpler logarithmic law, $\beta = 1$, is not supported by the available data.
