# Supplementary material for: Scaling properties of protein family phylogenies
Source: BMC Evol Biol. 2011 Jun 6;11:155. doi: 10.1186/1471-2148-11-155 (PMC3277297; doi:10.1186/1471-2148-11-155)
Supplement: Additional file 3 — Standard deviation of the evolvability model. Values of the standard error (SE) of the results from simulations of the evolvability model with respect to the PANDIT dataset, for values of p between [0.21 - 0.27]. A value p = 0.24 minimizes the error. [file 1471-2148-11-155-S3.PDF]

p	SE
0.27	0.23
0.26	0.24
0.25	0.2
0.24	0.18
0.23	0.23
0.22	0.26
0.21	0.3

Additional file 3 — Standard deviation of the evolvability model.
Values of the standard error (SE) of the results from simulations of the evolvability model with respect to the PANDIT dataset, for values of p between $[0.21 - 0.27]$. A value $p = 0.24$ minimizes the error.
